# Spin and charge drift-diffusion in ultra-scaled MRAM cells

**DOI:** 10.1038/s41598-022-25586-4

**Published:** 2022-12-05

**Authors:** Simone Fiorentini, Mario Bendra, Johannes Ender, Roberto L. de Orio, Wolfgang Goes, Siegfried Selberherr, Viktor Sverdlov

**Affiliations:** 1 Christian Doppler Laboratory for Nonvolatile Magnetoresistive Memory and Logic, Vienna, Austria; 2 Institute for Microelectronics, TU Wien, Gußhausstraße 27–29/E360, 1040 Vienna, Austria; 3 Silvaco Europe Ltd., Cambridge, UK

**Keywords:** Electrical and electronic engineering, Spintronics, Magnetic devices, Electronic and spintronic devices

## Abstract

Designing advanced single-digit shape-anisotropy MRAM cells requires an accurate evaluation of spin currents and torques in magnetic tunnel junctions (MTJs) with elongated free and reference layers. For this purpose, we extended the analysis approach successfully used in nanoscale metallic spin valves to MTJs by introducing proper boundary conditions for the spin currents at the tunnel barrier interfaces, and by employing a conductivity locally dependent on the angle between the magnetization vectors for the charge current. The experimentally measured voltage and angle dependencies of the torques acting on the free layer are thereby accurately reproduced. The switching behavior of ultra-scaled MRAM cells is in agreement with recent experiments on shape-anisotropy MTJs. Using our extended approach is absolutely essential to accurately capture the interplay of the Slonczewski and Zhang-Li torque contributions acting on a textured magnetization in composite free layers with the inclusion of several MgO barriers.

## Introduction

**Figure 1:**
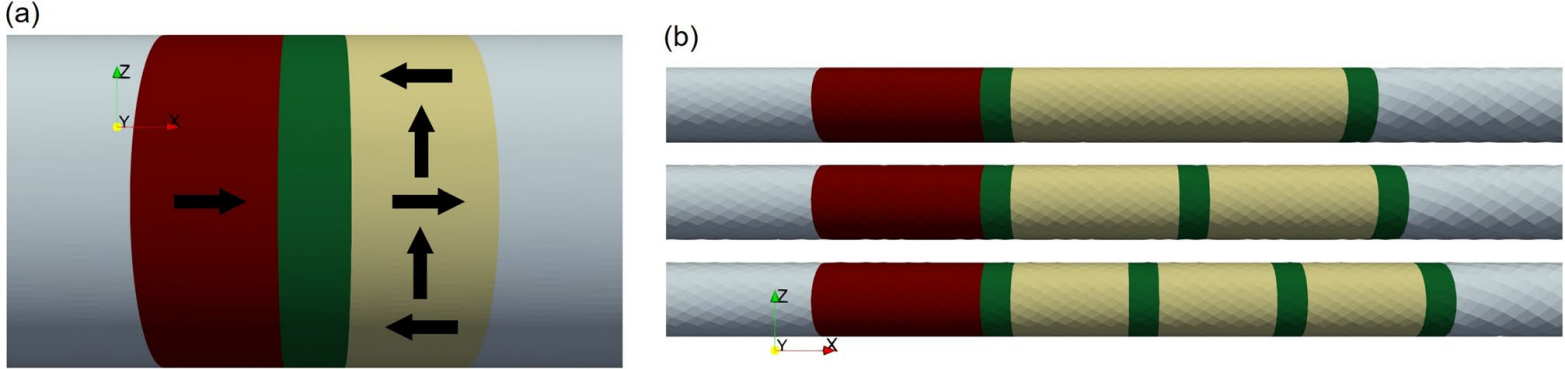
(**a**) MTJ structure with non-uniform magnetization configuration. The structure is composed of a reference layer (dark red), a tunnel barrier (green), a free layer (yellow), and two non-magnetic contacts (light blue). The arrows represent the magnetization orientation. (**b**) Model examples of elongated ultra-scaled MRAM cells, with single (top) or composite (middle and bottom) free layer.

The ever-improving semiconductor industry has relied, in recent years, on the down-scaling of its components. The presence of leakage currents has, however, caused an increase of the stand-by power consumption in traditional volatile memories like SRAM and DRAM^1^. Nonvolatile components would allow to avoid any stand-by power usage. Emerging nonvolatile spin-transfer torque (STT) magnetoresistive random access memory (MRAM) offers high speed and endurance and is attractive for stand-alone^2^, embedded automotive^3^, MCU, and IoT^4^ applications, as well as frame buffer memory^5^ and slow SRAM^6^. The core of an STT-MRAM cell consists of a magnetic tunnel junction (MTJ), cf. Fig. 1a, with two ferromagnetic layers separated by an oxide tunnel barrier (TB). The reference layer (RL) is fixed either by proper choice of materials or by antiferromagnetic pinning, while the magnetization of the free layer (FL) can be reversed. When the magnetization vectors in the two layers are parallel (P), the resistance is lower than in the anti-parallel state (AP), providing a way to store binary information. The percentage difference between the two resistance states is labeled tunneling magnetoresistance (TMR) ratio. In STT-MRAM, switching between the two stable configurations is achieved by running an electric current through the structure. The spin-polarization of the RL generates a spin current which, when entering the free layer, acts on the magnetization via the exchange interaction. When the magnetization vectors are not aligned, conservation of angular momentum causes the transverse spin current to be quickly absorbed, generating the spin-transfer torque^7,8^. Employing CoFeB for the ferromagnetic layers and MgO for the oxide layers allows to reach TMR values of up to 600%^9^. CoFeB and MgO also possess suitable properties for the fabrication of MTJs with perpendicular magnetic anisotropy (PMA), which present better thermal stability, better scalability, and a lower switching current^10^. In order to increase the interface PMA, provided by the MgO tunneling layer, the FL is often interfaced with a second MgO layer^11^. Recently, more advanced structures were proposed to boost the PMA even further, either by introducing more MgO layers in the FL or using the shape anisotropy of elongated FLs^12^, while also improving scalability thanks to a reduced diameter. Accurate simulation tools can provide valuable support in the design of these ultra-scaled MRAM cells, cf Fig. 1b. In order to model such devices, it is paramount to generalize the traditional Slonczewski^13^ approach for the torque computation, applicable only to thin FLs, to incorporate normal metal buffers or MgO barriers between multiple CoFeB free layers, as well as the barrier between the RL and FL, and the torques coming from magnetization textures or domain walls, which can be generated in elongated FLs. In this work, we present an extension of the drift-diffusion formalism for the computation of the torque in the presence of MTJs in the structure. The model is implemented in a finite element (FE) solver based on open-source software. We show how the proposed approach is able to reproduce the expected properties of the STT torque observed in MTJs. Moreover, we show how the STT contribution and the one coming from magnetization gradients in the bulk of the magnetic layers are non-additive, so that a unified treatment of the two contributions is necessary in order to describe the torque acting in the ultra-scaled MRAM devices. Finally, we present switching simulations carried out with the described approach. The parameters employed for all the simulations, unless specified differently in the text, are summarized in the supplementary material available online, together with the weak formulation employed by the FE solver.

## Model

In micromagnetic simulations, the magnetization dynamics is described by the Landau-Lifshitz-Gilbert equation:

1 $$\begin{aligned} \frac{\partial \textbf{m}}{\partial t} = -\gamma \mu _0 \textbf{m}\times \mathbf {H_{eff}}+\alpha \textbf{m}\times \frac{\partial \textbf{m}}{\partial t}+\frac{1}{M_S}\mathbf {T_S} \end{aligned}$$

$\textbf{m}$ is a unit vector pointing in the magnetization direction, $\gamma$ is the gyromagnetic ratio, $\mu _0$ is the magnetic permeability, $\alpha$ is the Gilbert damping constant, $M_S$ is the saturation magnetization, $\mathbf {H_{eff}}$ is an effective field containing the contribution of external field, exchange interaction, and demagnetizing field, and $\mathbf {T_S}$ is the STT term. We implemented the equation in a Finite Element (FE) solver based on the Open Source library MFEM^14^. The contribution of the demagnetizing field is evaluated only on the disconnected magnetic domain by using a hybrid approach combining the boundary element method and the FE method^15^. A complete description of the torque term, which allows to include all physical phenomena responsible for proper ultra-scaled MRAM operation, can be obtained by computing the non-equilibrium spin accumulation. For this purpose, the drift-diffusion (DD) formalism has already been successfully applied in a spin-valve structure with a non-magnetic spacer layer^16–18^. The drift-diffusion equations for charge and spin current density are^19^:

2a $$\begin{aligned} \mathbf {J_C}= & {} \sigma \textbf{E} + \beta _D D_e\frac{e}{\mu _B} \left[ \left( \nabla \textbf{S}\right) ^T\textbf{m}\right] \end{aligned}$$

2b $$\begin{aligned} \mathbf {\overline{J_S}}= & {} -\frac{\mu _B}{e} \beta _\sigma \sigma \textbf{m} \otimes \textbf{E}-D_e\nabla \textbf{S} \end{aligned}$$

$\mu _B$ is the Bohr magneton, *e* is the electron charge, $\beta _\sigma$ and $\beta _D$ are polarization parameters, $D_e$ is the electron diffusion coefficient, and $\textbf{E}$ is the electric field. $\mathbf {J_C}$ is the charge current density, $\mathbf {\overline{J_S}}$ is the spin polarization current density tensor, where the components $J_{S,ij}$ indicate the flow of the i-th component of spin polarization in the j-th direction, $\nabla \cdot \mathbf {\overline{J_S}}$ is the divergence of $\mathbf {\overline{J_S}}$ with components $\left( \nabla \cdot \mathbf {\overline{J_S}}\right) _i = \sum _j \frac{\partial J_{S,ij}}{\partial x_j}$, and $\nabla \textbf{S}$ is the vector gradient of $\textbf{S}$, with components $\left( \nabla \textbf{S}\right) _{ij} = \frac{\partial S_i}{\partial x_j}$. The term $\left( \nabla \textbf{S}\right) ^T\textbf{m}$ is a vector with components $\left( \left( \nabla \textbf{S}\right) ^T\textbf{m}\right) _i = \sum _j \frac{\partial S_j}{\partial x_i}m_j$. $\mathbf {\overline{J_S}}$ will be referred to as spin current density in the rest of the paper, and can be converted to the usual units by multiplying by $\hbar /(2\mu _B)$. By inserting the expression for $\textbf{E}$ obtained from (2a) in (2b), one obtains the expression for the spin current density:

3 $$\begin{aligned} \mathbf {\overline{J_S}}= & {} -\frac{\mu _B}{e} \beta _\sigma \textbf{m} \otimes \left( \mathbf {J_C}-\beta _D D_e\frac{e}{\mu _B} \left[ \left( \nabla \textbf{S}\right) ^T\textbf{m}\right] \right) -D_e\nabla \textbf{S} \end{aligned}$$

The spin accumulation in the steady-state can than be obtained readily:

4a $$\begin{aligned}{} & {} -\nabla \cdot \mathbf {\overline{J_S}}-D_e\frac{\textbf{S}}{\lambda _{sf}^2}-\mathbf {T_S}=\textbf{0} \end{aligned}$$

4b $$\begin{aligned}{} & {} \quad \mathbf {T_S}=-\frac{D_e}{\lambda _{J}^2}\textbf{m}\times \textbf{S}-\frac{D_e}{\lambda _{\varphi }^2}\textbf{m}\times \left( \textbf{m}\times \textbf{S}\right) \end{aligned}$$

$\lambda _{sf}$ is the spin-flip length, $\lambda _J$ is the spin exchange length, and $\lambda _{\varphi }$ is the spin dephasing length. The term $\mathbf {T_S}$ is the same one entering (1), as it describes the transfer of angular momentum between the magnetization $\textbf{m}$ and the spin accumulation $\textbf{S}$.

As the DD approach only accounts for semi-classical transport properties, it must be supplemented with appropriate conditions for the TB to account for the dependence of the torque on the tunneling process across the MTJ.

### Model extension to include MTJ properties

Through the NEGF formalism, it is possible to compute expressions for the charge and spin current flowing through the TB of an MTJ^20^. Such expressions can be simplified to include the most prominent characteristics of the transport in a few polarization parameters^21,22^:

5a $$\begin{aligned}{} & {} J_C^{TB} \backsim J_0(V)\,\left( 1+P_{RL}\,P_{FL}\,\cos \theta \right) \end{aligned}$$

5b $$\begin{aligned}{} & {} J_{S,x}^{TB} \backsim -\frac{{a_{mx}}\,P_{RL}+{a_{mx}}\,P_{FL}\,\cos \theta }{ 1+P_{RL}\,P_{FL}\,\cos \theta }\,\frac{\hbar }{2e}\,J_C^{TB} \end{aligned}$$

5c $$\begin{aligned}{} & {} J_{S,y}^{TB} \backsim -\frac{1/2\,\left( P_{RL}\,P_{RL}^\eta -P_{FL}\,P_{FL}^\eta \right) \,\sin \theta }{ 1+P_{RL}\,P_{FL}\,\cos \theta }\,\frac{\hbar }{2e}\,J_C^{TB} \end{aligned}$$

5d $$\begin{aligned}{} & {} J_{S,z}^{TB} \backsim -\frac{{a_{mx}}\,P_{FL}\,\sin \theta }{ 1+P_{RL}\,P_{FL}\,\cos \theta }\,\frac{\hbar }{2e}\,J_C^{TB} \end{aligned}$$

$J_0(V)$ contains the voltage-dependent portion of the current density, $P_{RL}$ and $P_{FL}$ are the in-plane Slonczewski polarization parameters, $P_{RL}^\eta$ and $P_{FL}^\eta$ are out-of-plane polarization parameters, and ${a_{mx}}$ describes the influence of the interface spin-mixing conductance on the transmitted in-plane spin current. The given expressions consider the RL magnetization pointing in the x-direction, and the FL magnetization lying in the xz-plane, at an angle $\theta$ with respect to the RL one.

We extended the DD approach to be able to include the above equations for the current flowing through the MTJ. We modeled the TB as a poor conductor with a local conductivity depending on the relative orientation of the magnetization^23^. The TB conductivity expression is:

6 $$\begin{aligned} \sigma \left( \mathbf {m_{RL}},\,\mathbf {m_{FL}}\right) = \sigma _0 \left( 1+ \left( P_{FL}\,P_{RL}\right) \mathbf {m_{RL}}\cdot \mathbf {m_{FL}}\right) \end{aligned}$$

$\sigma _0=(\sigma _P+\sigma _{AP})/2$ is the angle independent portion of the conductivity, $\sigma _{P(AP)}$ is the conductivity in the parallel (anti-parallel) state, and $\mathbf {m_{RL(FL)}}$ is the magnetization of the RL(FL) close to the interface. It is a manifestation of Ohm’s law relating the voltage and the charge current through a structure with many transversal modes^24^. Computing the TMR from (6) gives back the Julliere expression^25^:

7 $$\begin{aligned} TMR = \frac{G_P-G_{AP}}{G_{AP}} = \frac{2\,P_{RL}\,P_{FL}}{1-P_{RL}\,P_{FL}} \end{aligned}$$

$G_{P(AP)}$ is the conductance in the parallel (anti-parallel) state. To compute the current density, we solve:

8a $$\begin{aligned} -\nabla \cdot \left( \sigma \nabla V\right)= & {} 0 \end{aligned}$$

8b $$\begin{aligned} \mathbf {J_C}= & {} \sigma \nabla V \end{aligned}$$

**Figure 2:**
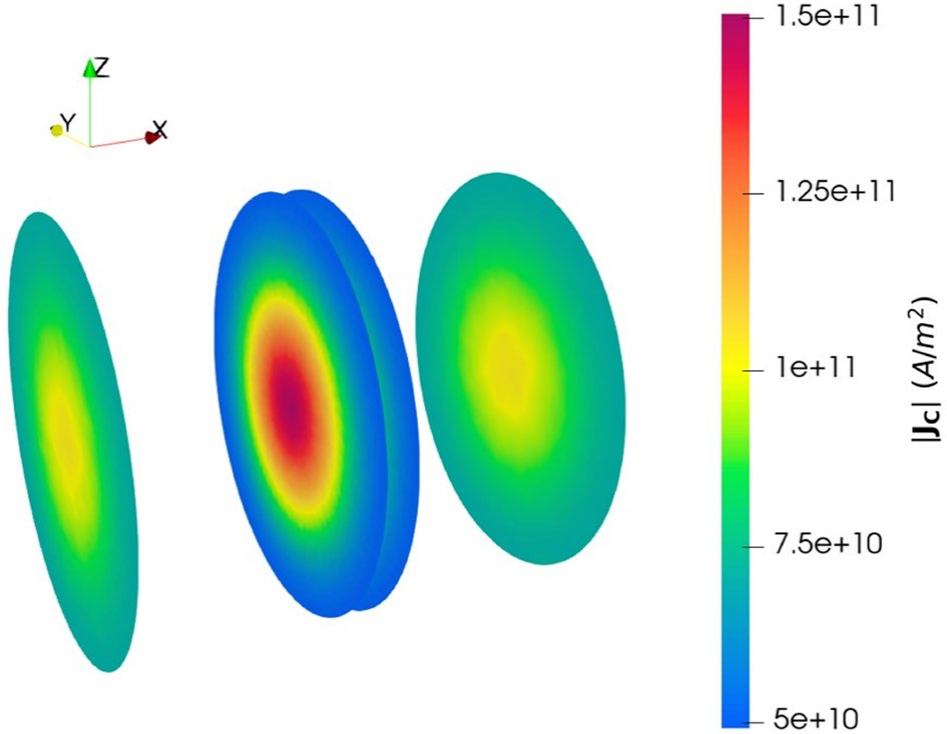
Current density in an MTJ biased under a constant voltage for the non-uniform FL magnetization configuration sketched in Fig. 1a. The center planes are at the TB interface, the side planes are in the NM contacts.

*V* is the elctrical potential and $\sigma$ is described by (6) in the tunnel barrier. Figure 2 shows the redistribution of the current density in an MTJ at a fixed voltage for the FL magnetization configuration shown in Fig. 1a. The structure has a diameter of 40 nm, the FL and RL are 2 nm thick, the TB is 1 nm thick, and the NM contacts are 50 nm thick. The current density is larger in the center, where the FL magnetization is parallel to that of the RL and the magnetization-dependent conductivity is the highest. The difference between lowest and highest current density values is dictated by TMR $\backsim$ 200%.

**Figure 3:**
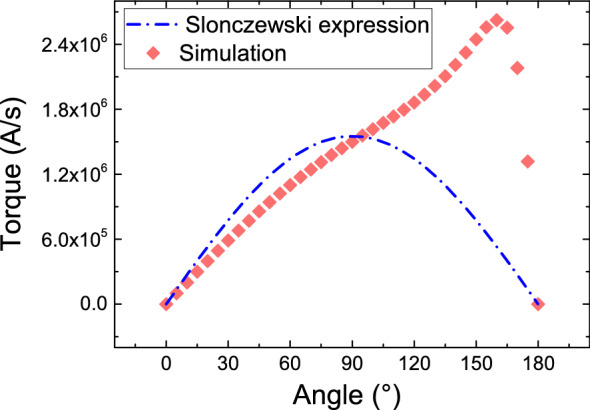
Angular dependence of the damping-like torque acting on a semi-infinite FL based on the DD equations (scatter plot) and on the Slonczewski expression^13^ (dash-dotted line).

**Figure 4:**
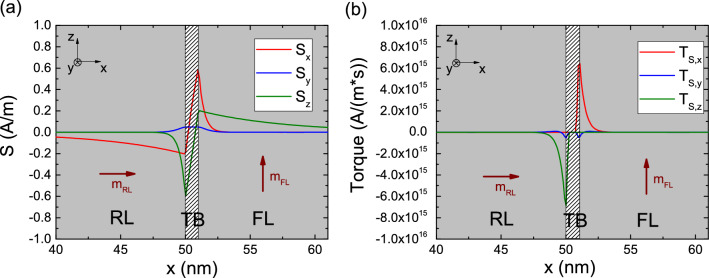
Spin accumulation (**a**) and torque (**b**) computed with the spin-current boundary condition (9) for an MTJ with semi-infinite ferromagnetic layers. Magnetization is along *x* in the RL and along *z* in the FL. The three curves represent x-, y-, and z-components of the computed spin accumulation and spin torque, respectively, along an axis going through the center of the structure. Brown vectors report the magnetization direction in both ferromagnetic layers.

While it is possible to use (3) and (4a) together with (6) to mimic the torque magnitude expected in an MTJ by tuning the tunnel barrier parameters^23^, some of the torque properties are not reproduced in this way. In Fig. 3, the angular dependence of the torque acting on a semi-infinite FL is compared with the Slonczewski expression^13^. The results show a clear deviation of the DD results from the expected ones. Therefore, the spin current part of () must also be accounted for. The traditional FE approach applied to the DD equations^16^ enforces the spin current and the spin accumulation to be continuous through all the interfaces. In order to include the spin current from equation () in the model, we take the diffusion coefficient of the TB to be low, proportionally to the conductivity, and apply the following expression as a boundary condition for both the RL|TB and TB|FL interface:

9 $$\begin{aligned} \mathbf {J_S^{TB}} = -\frac{\mu _B}{e} \, \frac{\mathbf {J_C^{TB}}\cdot \textbf{n}}{1 + P_{RL}\,P_{FL}\,\mathbf {m_{RL}}\cdot \mathbf {m_{FL}}} \left[ {a_{mx}}\,P_{RL}\,\mathbf {m_{RL}} + {a_{mx}}\,P_{FL}\,\mathbf {m_{FL}} + 1/2\,\left( P_{RL}\,P_{RL}^\eta - P_{FL}\,P_{FL}^\eta \right) \,\mathbf {m_{RL}}\times \mathbf {m_{FL}}\right] \end{aligned}$$

$\mathbf {J_C^{TB}}$ is the electric current density at the interface, $\textbf{n}$ is the interface normal, and $\mathbf {m_{RL(FL)}}$ is the unit magnetization vector of the RL(FL) at the interface. Doing this, we fix the spin current to the value prescribed by (9), when $\mathbf {J_C}$ flows through the MTJ. This is the key to describe the spin current and the spin accumulation in the RL and FL of an MTJ. Employing this approach gives the opportunity to describe the spin and charge transport coupled to the magnetization in arbitrary stacks of MTJs and metallic spin valves with a unified LLG-DD approach, and it allows to compute a fully three-dimensional solution in the presence of non-uniform magnetization configurations.

## Results and discussion

Our approach is applied to analyze spin accumulation and torque in a structure with semi-infinite ferromagnetic leads separated by a 1 nm thick tunnel junction, for uniform magnetization along x in the RL and along z in the FL. The results are shown in Fig. 4a and b. To evaluate $\mathbf {J_{S,TB}}$ with (9) at every boundary point of the RL|TB interface, with a magnetization value $\mathbf {m_{RL}}$, the solver looks for the closest point on the opposite TB|FL interface and uses its corresponding $\mathbf {m_{FL}}$ value. The same procedure is carried out for the TB|FL interface. The transverse spin dephasing length is $\lambda _\varphi =0.4$ nm, the exchange length is $\lambda _J=1$ nm, and the spin-flip length is $\lambda _{sf}=10$ nm. The short value of the dephasing length is employed to guarantee the fast absorption of the transverse components of the spin accumulation near the interface^26^, as expected in the presence of strong ferromagnets^13,27^. The boundary condition imposed by (9) creates a jump between the values of the spin accumulation components parallel to the magnetization at the left and right interface of the TB. This is the manifestation of the MTJ polarization effects on the spin current^28^. The transverse spin accumulation is quickly absorbed, so that the torques are acting near the interfaces. We note that computing the spin accumulation in the whole structure gives the torque acting in all the ferromagnetic layers from a unified expression.

**Figure 5:**
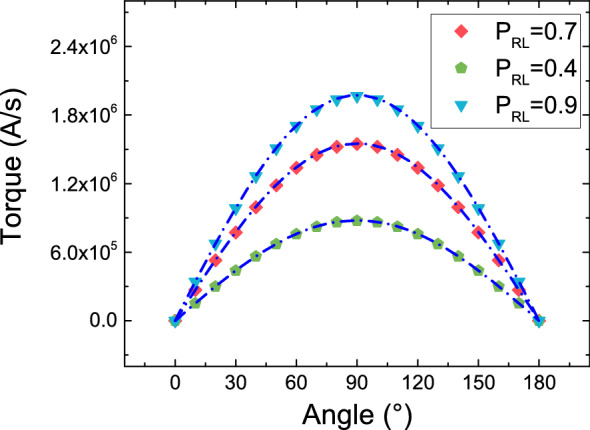
Angular dependence of the damping-like torque computed with the spin-current boundary conditions, for semi-infinite FL and RL. Dash-dotted lines represent the dependence described by the Slonczewski expression. The expected sinusoidal angular dependence of an MTJ is reproduced, for several values of the RL spin polarization parameter.

Figure 5 shows the angular dependence of the damping-like torque with the inclusion of the spin current boundary condition, for semi-infinite ferromagnetic layers. The typical sinusoidal dependence^13,21^ of the torque acting on the FL in an MTJ is now reproduced exactly, for various values of the RL|TB interface spin polarization. The structure is biased by a fixed voltage, so that the torque is independent of the TB|FL polarization, and only depends on the value of the RL|TB one.

**Figure 6:**
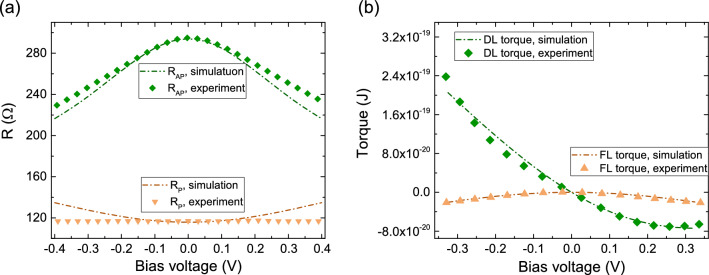
Dependence of both resistance (**a**) and damping-like (DL) and field-like (FL) torques (**b**) on the bias voltage, compared with experimental results^29^.

The implementation discussed until now produces a linear dependence of the torques on the bias voltage, with a vanishing damping-like component for $P_{RL}^\eta =P_{FL}^\eta$. Fabricated MRAM devices usually exhibit clear non-linearity in the observed bias dependence of both the torques and the TMR^29,30^. As a way to account for this non-linearity, bias dependence can be included in the polarization parameters $P_{RL}$ and $P_{FL}$. It can be postulated as^31^:

10 $$\begin{aligned} P_{RL}(V)=\frac{1}{1+P_0\,\exp (V/V_0)}, \,\,\, P_{FL}(V)=P_{RL}(-V) \end{aligned}$$

*V* is the voltage drop across the TB, $P_0$ can be extracted from the TMR at zero bias, and $V_0$ from the high bias behavior. A comparison of both TMR and torque results with experimental ones^29^ is reported in Fig. 6a and b, showing a good agreement. The results were obtained for $P_{RL}(0)=P_{FL}(0)=0.66$, $P_{RL}^\eta =P_{FL}^\eta =0.11$, ${a_{mx}}=0.36$, $V_0=0.65$ V, and $\sigma _0$ extracted from the anti-parallel resistance $R_{AP}=294$ $\Omega$ of the experimental structure, possessing a surface area of $70~\text {nm}\,\text {x}\,250~\text {nm}$^29^. Additional bias dependence features could be included by having $\sigma _0$ also depend on the applied bias voltage^32^.

### GMR effect in spin-valves

While the proposed approach is able to compute both the TMR and torque in an MTJ, in ultra scaled devices non-magnetic spacer layers can also be used to split the FL into two parts and avoid the formation of magnetization textures or domain walls. In a spin-valve with a metallic spacer layer, it is the Giant-Magnetoresistance (GMR) effect which causes the resistance of the structure to depend on the relative angle between the magnetization vectors. Such an effect can be accounted for by taking the magnetization-dependent contribution in (2a) into account when computing the current density. By taking $\nabla \cdot \mathbf {J_C}=0$ (in the absence of current sources) and $\textbf{E}=-\nabla V$ in (2a), one obtains the equation for the electrical potential.

11 $$\begin{aligned} -\nabla \cdot \left( \sigma \nabla V\right) = -\beta _D\,D_e\,\frac{e}{\mu _B}\nabla \cdot \left[ \left( \nabla \textbf{S}\right) ^T\textbf{m}\right] \end{aligned}$$

The additional right-hand side term depends on the spin accumulation, which in turn depends on the current density. In order to compute a solution which takes the interdependence into account, we iterate over the solution of (11) and (4a), until a convergence threshold is reached. This approach can be directly used for the FE implementation of the two separate equations, and does not require additional care for the inclusion of the boundary condition (9) in a coupled system of equations.

**Figure 7:**
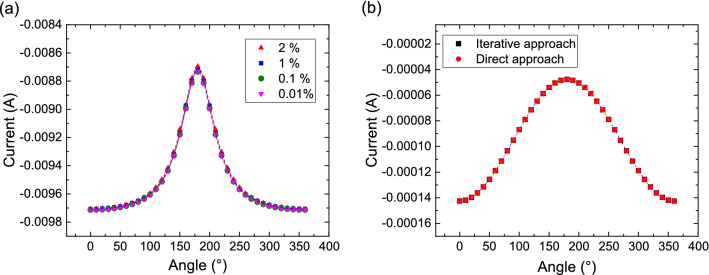
(**a**) Angular dependence of the total current in a spin-valve structure with metallic spacer, computed with the iterative approach for various values of the convergence parameter $\epsilon$. (**b**) Angular dependence of the total current in an MTJ, computed using both the direct and iterative approach.

The iterative solution is computed as follows:

1. We obtain a first estimate $\mathbf {S_0}$ of the spin accumulation by solving (8a) and (4a) with the spin current density taken from (3).
2. We use $S_0$ to compute the electrical potential from (11).
3. This potential is then used to obtain an updated solution $\mathbf {S_1}$ from (4a), with the spin current density now described by (2b).
4. Steps 2 and 3 are iterated until the solver reaches convergence.
  12 $$\begin{aligned} \frac{\Vert \mathbf {S_n}\Vert _{L2}-\Vert \mathbf {S_{n-1}}\Vert _{L2}}{\Vert \mathbf {S_n}\Vert _{L2}} < \epsilon \end{aligned}$$

Figure 7a shows the obtained dependence of the total current density on the relative angle between the magnetization vectors in the FL and RL, for several values of $\epsilon$. The solution is computed in the structure in Fig. 1a, with the middle layer treated as a non-magnetic metallic spacer, for an applied voltage of $-0.2$  V. The dashed lines represent a fit carried out using equation (13)^33^.

13 $$\begin{aligned} I(\theta ) = \frac{V}{R_P}\,\frac{1+\chi \,\cos ^2 \theta }{1+GMR+(\chi -GMR)\,\cos ^2 \theta } \end{aligned}$$

*V* is the applied bias voltage, $R_P$ is the resistance in the parallel state, and $\chi$ and *GMR* are used as fitting parameters. The obtained *GMR* is $\backsim 11\%$, with the results obtained using $\epsilon =1\%$ converging fast ($n\le 3$) and giving a good approximation.

Figure 7b reports the current dependence obtained by considering a tunneling middle layer. The data were computed both by using the direct solution of equations (2b), (4), (8) and the iterative solution described in this section. The fitting can be performed by using (6) as the angular dependence expression. As the iterative solver always converges for $n=1$ and the results are indistinguishable from the direct solution, these findings confirm that the latter can be safely employed for all structures only containing MTJs.

### Torques in elongated ultra-scaled devices

In the presence of elongated FLs like the ones in Fig. 1b, the switching of the whole layer at the same time is not guaranteed: a domain wall or magnetization textures can be generated, with their propagation through the FL affecting the switching behavior. In this case, the additional spin torques created by the presence of magnetization gradients in the bulk of the ferromagnetic layers must be taken into account. These torques are modeled by the Zhang and Li (ZL)^34^ equation. We generalized the ZL torques to include $\lambda _\varphi$ using the expression:

14 $$\begin{aligned} \mathbf {T_{ZL}} = -\frac{\mu _B}{e} \, \frac{\beta }{1+(\epsilon +\epsilon ')^2} \, \left( \left( 1+\epsilon '\,\left( \epsilon +\epsilon '\right) \right) \, \textbf{m}\times \left[ \textbf{m}\times \left( \mathbf {J_C}\cdot \nabla \right) \textbf{m} \right] - \epsilon \, \textbf{m} \times \left( \mathbf {J_C}\cdot \nabla \right) \textbf{m} \right) \end{aligned}$$

**Figure 8:**
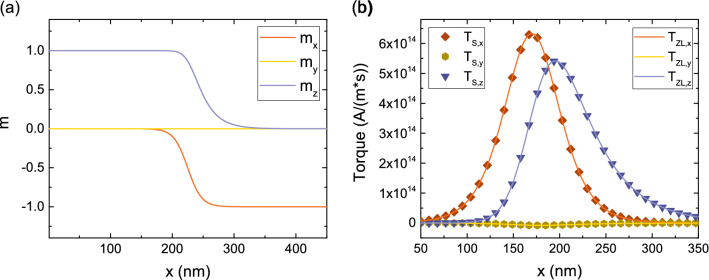
(**a**) Non-uniform magnetization texture with the magnetization orientation changing from z to -x. (**b**) Comparison of the spin torque $\mathbf {T_S}$ to the Zhang-Li torque $\mathbf {T_{ZL}}$ for a magnetization texture longer than $\lambda _{sf}$, for $\lambda _{J}=1$ nm and $\lambda _{\varphi }=5$ nm. The two approaches are in good agreement.

**Figure 9:**
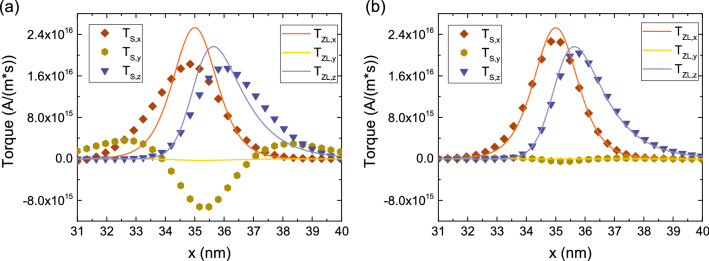
Comparison of the spin torque $\mathbf {T_S}$ to the Zhang-Li torque $\mathbf {T_{ZL}}$ for a magnetization texture shorter than $\lambda _{sf}$, for $\lambda _{J}=1$ nm and $\lambda _{\varphi } = 5$ nm in (**a**) and $\lambda _{\varphi }=0.4$ nm in (**b**). The shorter dephasing length takes the role of quickly absorbing the transverse spin accumulation components, so that the agreement between the two approaches is recovered.

$\epsilon = \left( \lambda _J / \lambda _{sf}\right) ^2$ and $\epsilon ' = \left( \lambda _J / \lambda _\varphi \right) ^2$. Such expression can be derived from the spin accumulation equation by taking $\nabla \textbf{S}=0$, and is strictly valid only when the change of magnetization in space happens over length scales longer than $\lambda _{sf}$. To test this assumption, we consider the magnetization profile shown in Fig. 8a, and compute the torque for $\lambda _{sf}=10$ nm, $\lambda _{J}=1$ nm, $\lambda _{\varphi }=5$ nm. Figure 8b demonstrates that, for a magnetization profile width of $\backsim 100$ nm, $\mathbf {T_S}$ is well reproduced with (14). However, if the width of the magnetization profile is reduced to $\backsim 3$ nm, the spin accumulation gradients neglected in (14) affect the result, and a large deviation of $\mathbf {T_S}$ from $\mathbf {T_{ZL}}$ is observed, especially for the field-like torque (y-component), as shown in Fig. 9a. However, the presence of a short spin dephasing length, $\lambda _\varphi =0.4$ nm, guarantees the fast absorption of the transverse spin, and a good agreement between $\mathbf {T_S}$ and $\mathbf {T_{ZL}}$ is recovered, cf. Fig. 9b.

In MRAM cells with elongated FLs the MTJ and ZL torque contribution act at the same time in the presence of magnetization textures and domain walls in the bulk of the layer. We compute the torque in an experimental MTJ structure^12^ with a 5 nm RL, 0.9 nm TB, and an elongated FL of 15 nm with a magnetization profile in the FL similar to the one shown in Fig. 8a, with the magnetization vector going from the z-direction to the -x-direction over the length of the layer. The magnetization in the RL is pointing towards the x-direction. The solution is computed with the same parameters as the ones employed for Fig. 9b.

**Figure 10:**
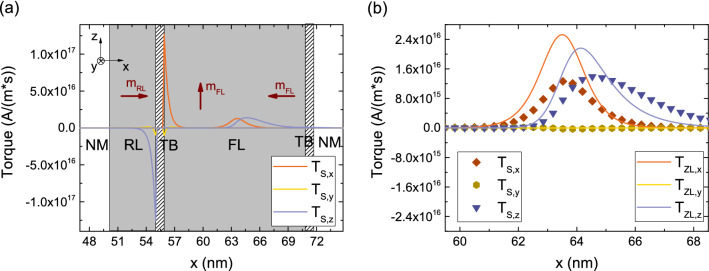
(**a**) Torques computed for an MRAM cell with elongated RL and FL and a magnetization profile in the FL similar to the one of Fig. 8a. The brown vectors indicate the magnetization direction in the RL and in two parts of the FL. (**b**) Close-up of the spin torque $\mathbf {T_S}$ compared to the Zhang-Li torque $\mathbf {T_{ZL}}$. The presence of the MTJ influences also the bulk portion of the torque, making the unified approach the most suitable for dealing with ultra-scaled MTJs with elongated ferromagnetic layers.

The torque $\mathbf {T_S}$ acting in the FL for this magnetization profile is shown in Fig. 10a. Both the interface contribution from the TB and the bulk ZL contribution are present. In Fig. 10b we show a close-up of the bulk portion of $\mathbf {T_S}$, compared with the ZL torque $\mathbf {T_{ZL}}$ computed in the FL for the same magnetization configuration. The comparison reveals a substantial difference between the torques obtained with our model and the traditional approach, where the ZL torque is simply added to the Slonczewski term, even in the presence of a short spin dephasing length. Our approach clearly demonstrates that, in an MTJ with elongated ferromagnetic layers, the Slonczewski and ZL torques are not independent: the presence of the TB also generates a spin accumulation component parallel to the magnetization, whose decay is dictated by $\lambda _{sf}$, cf. Fig. 4a. This component interacts with the magnetization texture, modifying the ZL torque contribution. A unified treatment of the MTJ polarization process and FL magnetization texture is thus required to accurately describe the torque and switching in ultra-scaled MRAM.

**Figure 11:**
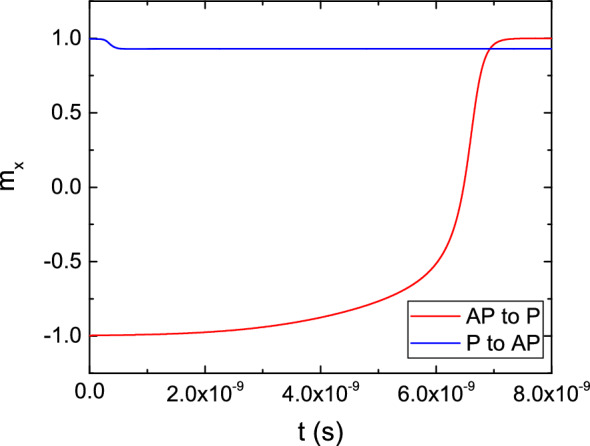
Switching results for a structure with an elongated FL, for both the AP to P and P to AP scenarios, under a bias voltage of 1.5 V.

**Figure 12:**
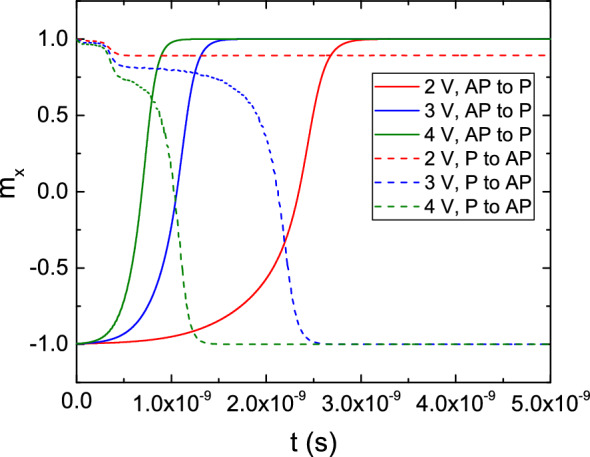
Switching results for a structure with an elongated FL under increasing bias voltage values, for both the AP to P and P to AP scenarios.

**Figure 13:**
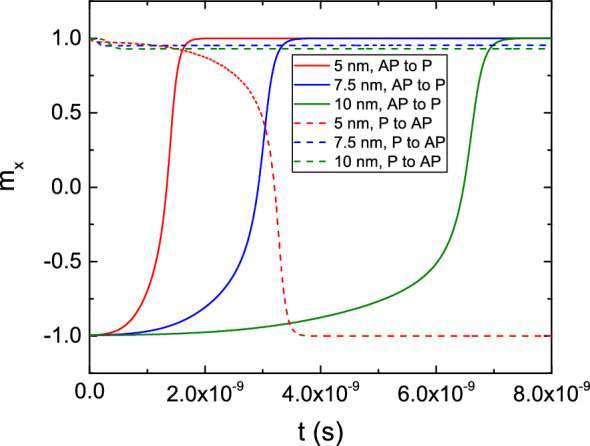
Switching results for a structure with an elongated FL for several lengths of the FL.

Finally, we investigate the magnetization behavior during switching in ultra-scaled MRAM cells with a diameter of 2.3 nm recently experimentally demonstrated^12^. The values of the resistance-area product (RA) and the TMR in the simulated structures are 2 $\Omega \,\mu \text {m}^2$ and 100%, respectively. In Fig. 11 the behavior of the top cell of Fig. 1b, with a single FL of 10 nm length, capped by an MgO TB separating it from the non-magnetic contact, is presented, under a bias voltage of 1.5 V. The thickness of the RL, TBs, and non-magnetic contacts are 5 nm, 0.9 nm, and 50 nm, respectively. The magnetization of the RL is in the positive x-direction. The magnetization of the FL is tilted $5^\circ$ away from the perfect P or AP orientation, to emulate the destabilizing effect of a non-zero temperature on the system. The precise value employed for the tilting angle only affects the duration of the incubation period before the start of the switching process, and doesn’t change the overall behavior of the magnetization reversal. The value of the bias voltage, while being sufficient to achieve switching for the AP to P scenario, is not enough to reverse the magnetization from P to AP. The additional stability of the parallel configuration comes from the stray field contribution of the RL, which favors it. Due to the presence of a stronger spin accumulation component parallel to the magnetization at the TB interface in the P state, the interaction of the Slonczewski and Zhang-Li torque contributions quickly generates a texture in the magnetization, whose average x-component slightly deviates from the starting configuration, as evidenced by the dip in the plot during the first nanoseconds of the simulation. Despite this, the overall torque is not strong enough to overcome the perpendicular anisotropy. We carried out additional simulations with bias values from 2 to 4  V, presented in Fig. 12, showing how an increased bias voltage, which entails an increased value of the torque, is able to achieve switching for both configurations. Moreover, we investigated the switching behavior of structures with FL thickness of 5 nm and 7.5 nm. The results are presented in Fig. 13. A shorter layer possesses a reduced energy barrier separating the two magnetization configurations^35^, so that the speed of AP to P switching is improved, and P to AP switching is achieved in the case of the 5 nm layer.

**Figure 14:**
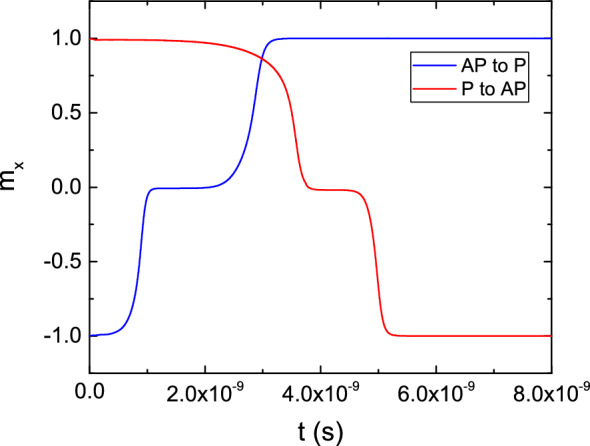
Switching results for a structure with composite FL, for both the AP to P and P to AP scenarios, under a bias voltage of 1.5 V.

**Figure 15:**
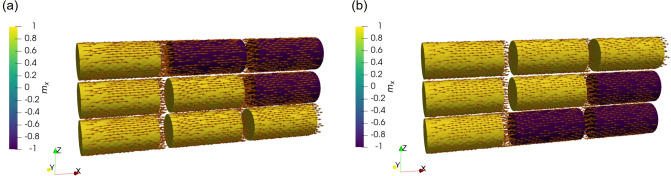
Switching stages of an ultra-scaled STT-MRAM cell with composite free layer, showcasing how the different parts of the FL switch one at a time. The RL is the first section on the left of the structure, while the second and third sections are the two parts of the FL (from left to right, FL1 and FL2, respectively). AP to P switching is presented in (**a**), while P to AP switching in (**b**).

The switching performance can be improved by employing a structure where the FL is split into two parts of 5 nm length by an additional MgO layer in the middle, presented in the middle of Fig. 1b. The addition of an MgO layer boosts the stability of the composite FL because of an increased interface anisotropy contribution, while the two parts of the FL have a preferred aligned configuration because of the stray field they exert on one another^12^. We employed our approach to carry out switching simulations in such a structure, under a bias voltage of 1.5 V, presented in Fig. 14. The resulting plot evidences how the switching process is overall faster in the composite structure as compared to the one with a single elongated layer, and that P to AP switching is achieved for lower values of the bias voltage. Figure 15 shows how the improved performance of the second structure comes from the composite nature of the FL, allowing for the different sections of the FL layer to be switched one at a time. In the AP configuration, the RL exerts a torque on the first part of the FL (FL1) to push it in the positive x-direction, parallel to it. At the same time, the second part of the FL (FL2) also exerts a torque on FL1 to push the magnetization to the positive x-direction, so that it is anti-parallel to FL2. Both torques’ contributions act in the same direction, causing FL1 to switch first and fast. At the same time, the torque acting from FL1 towards FL2 favors the two magnetization vectors to be parallel, keeping FL2 in its original orientation. However, after the magnetization of FL1 has switched, the torque acting on FL2 changes its sign, forcing it to switch. As the torque acts only from FL1, the magnitude is smaller than that acting in the first part of the switching process, resulting in a slower reversal of the FL2 magnetization. The three stages of AP to P switching are showcased in Fig. 15a. When going from P to AP, the opposite process happens. The torque acting from FL2 on FL1 is opposite to that from the RL, while the torque acting from FL1 on FL2 is favoring magnetization reversal, so that FL2 switches first. As only the torque from FL1 is acting, the switching time of FL2 is relatively slow. After FL2 has switched, the torque contributions from FL2 and the RL act on FL1 in the same direction, completing the switching fast. The three stages of P to AP switching are shown in Fig. 15b. The obtained switching time and the applied bias voltage agree well with the experimentally reported results^12^, and show how our approach can be employed to investigate the switching behavior of MRAM devices.

**Figure 16:**
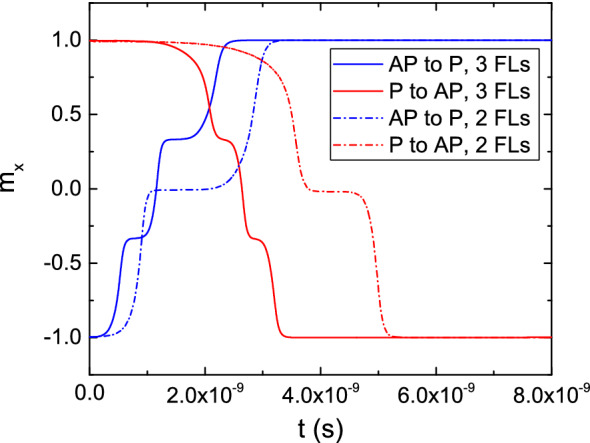
Switching results for a structure with three FL segments, for both the AP to P and P to AP scenarios, under a bias voltage of 1.5 V (solid line). The magnetization trajectories are compared to the ones obtained in the structure with two FL segments (dash-dotted lines).

In order to further analyze the performance of a composite FL, we performed simulations to investigate the behavior of a structure with three FL segments of 3.5 nm length each, to keep the same aspect ratio of the whole FL. The structure is reported on the bottom of Fig. 1b. All the segments are separated by 0.9 nm thick MgO layers. The results for both AP to P and P to AP switching are reported in Fig. 16, for a bias voltage of 1.5 V. The switching process is qualitatively similar to the one of the structure with two FL segments, with the three sections of the FL switching one at a time. In the AP configuration, the torque coming from the RL and FL2 causes the fast switching of FL1 to the positive x-direction. At this point, the torque coming from FL1 and the third part of the FL (FL3) causes FL2 to also switch fast towards the positive x-direction. Finally, as only the torque coming from FL2 acts on FL3, the latter has a slower magnetization reversal which completes the switching process. When going from P to AP, as is the case for the structure with two FL segments, the opposite process happens. The torques acting on FL1 and FL2 from the adjacent layers compensate each other, and only the torque acting from FL2 on FL3 is able to cause the magnetization reversal of the latter. At this point, the torque acting from FL1 and FL3 on FL2 becomes additive, so that FL2 switches faster. This is finally followed by the fast switching of FL1, as the torque contributions coming from the RL and FL2 push its magnetization towards the negative x-direction. As shown in Fig. 16, the complete switching process is faster in the structure with three FL segments, for both AP to P and P to AP realizations. This indicates that increasing the number of segments provides an advantage in terms of switching time and bias, and the multiple magnetization states reached during the switching process make these structures promising candidates as multi-bit memory cells.

## Conclusion

We presented a modeling approach to accurately describe the charge and spin currents, the torques, and the magnetization dynamics in ultra-scaled MRAM cells consisting of several elongated pieces of ferromagnets separated by multiple tunnel barriers. We showed how the fully 3D spin and charge drift-diffusion equations can be supplied with appropriate conditions at the tunneling layer to reproduce the TMR effect as well as the angular and voltage dependence of the torque expected in MTJs. We reported how an iterative solution of the charge and spin accumulation equations can be employed to account for the GMR effect. The advantage of the proposed approach is the possibility of computing all the torque contributions from a unified expression, so that the interactions between them can be evaluated, and the torque acting in the presence of multiple layers of varying thickness is automatically accounted for, even for non-uniform magnetization distributions. We demonstrated that the Slonczewski and Zhang and Li torques are not additive and must be derived from the spin accumulation to account for their interplay and correctly describe the torques on textured magnetization in elongated FLs with several MgO TBs. Finally, we applied the presented method to switching simulations of MRAM cells with elongated and composite FLs. The obtained results validate the use of the proposed simulation approach as support for the design of advanced ultra-scaled MRAM cells.

## Supplementary Information

Supplementary Information.

## Data Availability

The datasets generated during and/or analyzed during the current study are available from the corresponding author on reasonable request.
