# Supplementary material for: Spin and charge drift-diffusion in ultra-scaled MRAM cells
Source: Sci Rep. 2022 Dec 5;12:20958. doi: 10.1038/s41598-022-25586-4 (PMC9723118; doi:10.1038/s41598-022-25586-4)
Supplement: Supplementary file 1 — Supplementary Information. [file 41598_2022_25586_MOESM1_ESM.pdf]

Spin and Charge Drift-Diffusion in Ultra-Scaled MRAM Cells

Simone Fiorentini[1,2,*], Mario Bendra[1,2], Johannes Ender[1,2], Roberto L. de Orio[1,2], Wolfgang Goes[3], Siegfried Selberherr[2], and Viktor Sverdlov[1,2]

[1]Christian Doppler Laboratory for Nonvolatile Magnetoresistive Memory and Logic at the
[2]Institute for Microelectronics, TU Wien, Gußhausstraße 27–29/E360, 1040 Vienna, Austria
[3]Silvaco Europe Ltd., Cambridge, United Kingdom
[*]fiorentini@iue.tuwien.ac.at

SUPPLEMENARY INFORMATION

Simulation Parameters and Weak Formulation

The parameters employed for computing the spin accumulation, unless differently specified in the text, are reported in Table S1. The parameters employed for the LLG equation are reported in Table S2. The considered interface anisotropy coefficient for each tunnel barrier contacting a given ferromagnetic layer is K_i=1.53 J/m^2. The uniaxial anisotropy coefficient K employed in the LLG equation is obtained from it by multiplying by the number of tunnel barriers contacting the layer and dividing by its length. The value reported in Table S2 is the one employed for the free layer (FL) segments of the composite FL structure reported in the middle of Fig. 1 in the main manuscript.

**Parameter**	**Value**
Charge polarization, β_σ	0.7
Spin polarization, β_D	0.8
NM electron diffusion coefficient, $D_{e,NM}$	10^{-2} m^2/s
FL and RL electron diffusion coefficient, $D_{e,FM}$	2.0×10^{-3} m^2/s
TB electron diffusion coefficient, $D_{e,TB}$	2.0×10^{-8} m^2/s
Spin flip length, λ_{sf}	10 nm
Spin exchange length, λ_J	1 nm
Spin dephasing length, λ_φ	0.4 nm
NM conductivity, σ_{NM}	5.0×10^6 S/m
FM conductivity, σ_{FM}	1.0×10^6 S/m
TB conductivity, σ_0	29.76 S/m
Polarization factors $P_{RL}(0)=P_{FL}(0)$	0.707
In-plane torque reduction a_{mx}	1.0
Out-of-plane polarization $P_{RL}^\eta=P_{FL}^\eta$	0.2

Table S1. Parameters used in the DD simulations.

**Parameter**	**Value**
Saturation magnetization, M_S	1.2×10^6 A/m
Exchange coefficient, A_{ex}	10^{-11} J/m
Anisotropy coefficient, K	6.12×10^5 J/m^3
Gilbert damping, α	0.02

Table S2. Parameters used in the LLG simulations.

The weak formulation of equation (1) in the main manuscript, employed by the FE solver, is:

$$\mathbf{v} = \partial_t \mathbf{m}^k, \qquad \mathbf{v}\cdot\mathbf{m}^k = 0$$
$$\int_\omega \left(\alpha\mathbf{v} + \mathbf{m}^k\times\mathbf{v}\right)\cdot\mathbf{w}d\mathbf{x} + \theta\frac{2A_{ex}\gamma}{M_S}\delta t\int_\omega \nabla\mathbf{v}:\nabla\mathbf{w}d\mathbf{x} =$$
$$\gamma\mu_0\int_\omega \mathbf{H_{eff}}\cdot\mathbf{w}d\mathbf{x} - \frac{2A_{ex}\gamma}{M_S}\int_\omega \nabla\mathbf{m}:\nabla\mathbf{w}d\mathbf{x} + \int_\omega\left(\frac{D_e}{M_S\lambda_J^2}\mathbf{S^k} + \frac{D_e}{M_S\lambda_\varphi^2}\mathbf{m^k}\times\mathbf{S^k}\right)\cdot\mathbf{w}d\mathbf{x}$$
$$\mathbf{m^{k+1}} = \frac{\mathbf{m^k}+\delta t\,\mathbf{v}}{|\mathbf{m^k}+\delta t\,\mathbf{v}|} \tag{S1}$$

The weak formulation of equation (4) in the main manuscript is:

$$D_e\int_\Omega \nabla\mathbf{S}:\nabla\mathbf{w}d\mathbf{x} - D_e\beta_\sigma\beta_D\int_\Omega\left[\mathbf{m}\otimes\left((\nabla\mathbf{S})^\mathbf{T}\mathbf{m}\right)\right]:\nabla\mathbf{w}d\mathbf{x} + \frac{D_e}{\lambda_{sf}^2}\int_\Omega \mathbf{S}\cdot\mathbf{w}d\mathbf{x} + \frac{D_e}{\lambda_J^2}\int_\Omega(\mathbf{S}\times\mathbf{m})\cdot\mathbf{w}d\mathbf{x}$$
$$+\frac{D_e}{\lambda_\varphi^2}\int_\Omega(\mathbf{m}\times(\mathbf{S}\times\mathbf{m}))\cdot\mathbf{w}d\mathbf{x} = \frac{\mu_B}{e}\beta_\sigma\int_\omega[\mathbf{m}\otimes\mathbf{J_C}]:\nabla\mathbf{w}d\mathbf{x} - \frac{\mu_B}{e}\beta_\sigma\int_{\partial\Omega\cap\partial\omega}([\mathbf{m}\otimes\mathbf{J_C}]\,\mathbf{n})\cdot\mathbf{w}d\mathbf{x}$$
$$+\int_{RL|TB} -\mathbf{J_{S,TB}}\cdot\mathbf{w}d\mathbf{x} + \int_{TB|FL}\mathbf{J_{S,TB}}\cdot\mathbf{w}d\mathbf{x} \tag{S2}$$

$\mathbf{w}$ is a vector test function, Ω is the whole volume of the structure, ω is the volume of the magnetized regions, and $\mathbf{n}$ is the boundary outer normal. $\nabla\mathbf{a}:\nabla\mathbf{b} = \sum_{ij}(\nabla\mathbf{a})_{ij}(\nabla\mathbf{b})_{ij}$ is the Frobenius inner product of two matrices. $\mathbf{J_{S,TB}}$ is defined by Equation (9) in the main text, and $RL|TB\,(TB|FL)$ indicates the interface between a TB and the RL (FL). The Neumann condition $\frac{\partial\mathbf{S}}{\partial\mathbf{n}} = \mathbf{0}$ is assumed on external boundaries.

The weak formulation of equation (8a) in the main manuscript is:

$$\int_\Omega \sigma(\theta)\nabla V\cdot\nabla v\,d\mathbf{x} = 0 \tag{S3}$$

v is a scalar test function. Dirichlet conditions are applied to prescribe the voltage at the left and right boundaries of the structure. The Neumann condition $\frac{\partial V}{\partial\mathbf{n}} = 0$ is assumed on external boundaries.

For the iterative solution, after the first spin accumulation estimate is computed, the weak formulation of the electric potential equation (11) in the main manuscript is:

$$\int_\Omega \sigma(\theta)\nabla V\cdot\nabla v\,d\mathbf{x} = \int_\omega \beta_D D_e\frac{e}{\mu_B}\left[(\nabla\mathbf{S})^T\mathbf{m}\right]\cdot\nabla v\,d\mathbf{x} \tag{S4}$$

The one for the spin accumulation is:

$$D_e\int_\Omega \nabla\mathbf{S}:\nabla\mathbf{w}d\mathbf{x} + \frac{D_e}{\lambda_{sf}^2}\int_\Omega \mathbf{S}\cdot\mathbf{w}d\mathbf{x} + \frac{D_e}{\lambda_J^2}\int_\Omega(\mathbf{S}\times\mathbf{m})\cdot\mathbf{w}d\mathbf{x}$$
$$+\frac{D_e}{\lambda_\varphi^2}\int_\Omega(\mathbf{m}\times(\mathbf{S}\times\mathbf{m}))\cdot\mathbf{w}d\mathbf{x} = \frac{\mu_B}{e}\beta_\sigma\int_\omega[\mathbf{m}\otimes\sigma\mathbf{E}]:\nabla\mathbf{w}d\mathbf{x} - \frac{\mu_B}{e}\beta_\sigma\int_{\partial\Omega\cap\partial\omega}([\mathbf{m}\otimes\sigma\mathbf{E}]\,\mathbf{n})\cdot\mathbf{w}d\mathbf{x}$$
$$+\int_{RL|TB} -\mathbf{J_{S,TB}}\cdot\mathbf{w}d\mathbf{x} + \int_{TB|FL}\mathbf{J_{S,TB}}\cdot\mathbf{w}d\mathbf{x} \tag{S5}$$
